# Cloning and Functional Analysis of Histones H3 and H4 in Nuclear Shaping during Spermatogenesis of the Chinese Mitten Crab, *Eriocheir sinensis*

**DOI:** 10.1371/journal.pone.0126623

**Published:** 2015-05-19

**Authors:** Jiang-Li Wu, Xian-Jiang Kang, Ming-Shen Guo, Shu-Mei Mu, Zhao-Hui Zhang

**Affiliations:** College of Life Sciences, Hebei University, Baoding, 071002, China; University of Alberta, CANADA

## Abstract

During spermatogenesis in most animals, the basic proteins associated with DNA are continuously changing and somatic-typed histones are partly replaced by sperm-specific histones, which are then successively replaced by transition proteins and protamines. With the replacement of sperm nuclear basic proteins, nuclei progressively undergo chromatin condensation. The Chinese Mitten Crab (*Eriocheir sinensis*) is also known as the hairy crab or river crab (phylum Arthropoda, subphylum Crustacea, order Decapoda, and family Grapsidae). The spermatozoa of this species are aflagellate, and each has a spherical acrosome surrounded by a cup-shaped nucleus, peculiar to brachyurans. An interesting characteristic of the *E*. *sinensis* sperm nucleus is its lack of electron-dense chromatin. However, its formation is not clear. In this study, sequences encoding histones H3 and H4 were cloned by polymerase chain reaction amplification. Western blotting indicated that H3 and H4 existed in the sperm nuclei. Immunofluorescence and ultrastructural immunocytochemistry demonstrated that histones H3 and H4 were both present in the nuclei of spermatogonia, spermatocytes, spermatids and mature spermatozoa. The nuclear labeling density of histone H4 decreased in sperm nuclei, while histone H3 labeling was not changed significantly. Quantitative real-time PCR showed that the mRNA expression levels of histones H3 and H4 were higher at mitotic and meiotic stages than in later spermiogenesis. Our study demonstrates that the mature sperm nuclei of *E*. *sinensis* contain histones H3 and H4. This is the first report that the mature sperm nucleus of *E*. *sinensis* contains histones H3 and H4. This finding extends the study of sperm histones of *E*. *sinensis* and provides some basic data for exploring how decapod crustaceans form uncondensed sperm chromatin.

## Introduction

Histones comprise a group of basic proteins that are responsible for DNA packing and chromatin condensation in the nuclei of almost all eukaryotic cells (excluding mature mammalian spermatozoa or dinoflagellates). Five histone types have been identified: H1 (or its variants), H3, H4, H2A, and H2B. Their encoding genes have no introns, and their transcripts are not poly A tailed [1–4]. Among them, histones H3 and H4 are the most conservative forms, with only one amino acid difference between the sea urchin and bovine H3 types, and only two amino acid differences out of 102 residues between the pea and bovine versions of H4.

Spermatogenesis is the process of developing immature germ cells known as spermatogonia into mature spermatozoa. The progression from spermatid to spermatozoon is called spermiogenesis. The basic proteins associated with DNA undergo continuous change throughout spermatogenesis of most animal species: somatic-typed histones are partly replaced by sperm-specific histones, which are then successively replaced by transition proteins and protamines. With the replacement of sperm nuclear basic proteins (SNBPs), the appropriate interaction of histones or SNBPs with DNA leads to the progressive condensation of sperm chromatin. As a result, the final sperm nucleus contains very compact chromatin, and the protein constitution is simplified. However, in one group of decapod crustaceans the mature sperm nuclei differ markedly from those in other taxa in which the chromatin has a decondensed, loosely arranged organization [5–8]. This characteristic is quite different from the sperm nuclei of other animals, which have highly condensed chromatin, possibly to protect the DNA from being mutated by environmental factors [9, 10].

Because spermatogenesis in many decapod species involves decondensation of the sperm chromatin, many researchers have focused on analyzing the SNBPs in these organisms. Previous studies, which were designed to determine the histone contents of the nucleus of crustacean crabs, mainly used histochemical staining methods [11]. Chevaillier (1966, 1967, 1968) described that histones migrated from the nucleus to the acrosome in *Eupagurus bernhardus* and *Carcinus maenas* [12–14]. Vaughn and Locy (1969) reported that large quantities of nucleoproteins in spermatids of *Emerita analoga* were reduced gradually during spermatogenesis, and that by the late stages there were nearly no histones nor protamines in sperm nuclei [15]. Kleve *et al* (1980) found that there were no basic proteins in sperm nuclei of *Sicyonia ingentis* [16]. Therefore, earlier researchers reported that these decapod crustacean sperm nuclei did not contain basic proteins, and that their chromatin was uncondensed [17]. However, recent studies on *Cancer pagurus* and *Cancer magister* [18], *Maja brachydactyla* [19] and *Portunus pelagicus* [20] provided new evidence regarding the longstanding question of histones in mature crustacean spermatozoa. These sperm nuclei contain relatively low contents of whole or parts of histones.

Because there is great interest in understanding the distribution of histones and exploring the reason for uncondensed chromatin in crustacean sperm, we have examined the most conserved histones (H3 and H4) expression profiles during spermatogenesis of *Eriocheir sinensis*. As several other crab species have been used in earlier studies, this particular species is an interesting model. The Chinese Mitten Crab, also known as the hairy crab or the river crab, belongs taxonomically to the phylum Arthropoda, subphylum Crustacea, order Decapoda, and family Grapsidae. It is an important commercial aquatic species in China.

In this study, we cloned the gene sequences encoding histones H3 and H4 and reported their expression pattern during spermatogenesis of *E*. *sinensis*, using immunofluorescence and immunoelectron microscopy. We found that histones H3 and H4 are present in the nuclei of spermatogonia, spermatocytes, spermatids and mature spermatozoa. In addition, we also analyzed the mRNA expression levels of histones H3 and H4 during spermatogenesis. The results showed that not all histones are lost during spermatogenesis. This is the first report that the mature sperm nucleus of *E*. *sinensis* contains histones H3 and H4.

## Materials and Methods

Immature male *E*. *sinensis* (in their rapid development phase) and mature male *E*. *sinensis* were purchased from Baiyangdian Lake, Hebei Province, P. R. China. The animals were anesthetized by chilling on ice, and then were dissected immediately to obtain testes, sperm ducts and seminal vesicles. No official approval is needed for the study of this crab in China.

### Antibodies

Rabbit anti-histone H3 polyclonal antibody (ab1791), mouse anti-histone H4 monoclonal antibody (ab31830), goat polyclonal secondary antibody to rabbit IgG-Fc (DyLight 488) (ab98462), goat anti-mouse IgG H&L (Cy3) (ab97035), goat anti-rabbit IgG H&L (10 nm Gold) (ab39601), and goat anti-mouse IgG H&L (20 nm Gold) (ab27242) were purchased from Abcam (Cambridge, MA, USA). Horseradish peroxidase (HRP)-conjugated goat anti-rabbit, and HRP-conjugated goat anti-mouse antibodies were purchased from Beijing Solarbio Science&Technology Co., Ltd. (Beijing, P. R. China).

### Polymerase chain reaction (PCR) amplification, cloning and sequencing of the H3 coding sequence in *E*. *sinensis*

DNAiso reagent (Takara, Dalian, P. R. China) was used to extract genomic DNA from testes according to the manufacturer’s protocol. Then we compared the full-length H3 amino acid sequences of *Homo sapiens* (GenBank accession No. AAN10053.1), *Mus musculus* (AAA37811.1), and partial H3 amino acid sequences of *Pachygrapsus transversus* (CBA13078.1), *Grapsus grapsus* (CBA13077.1), *Pachygrapsus marmoratus* (AAZ39265.1), which also belong to the Decapoda. It has been shown that the H3 amino acid sequences of *H*. *sapiens* and *M*. *musculus* are identical, as are the partial H3 amino acid sequences of *P*. *transversus*, *G*. *grapsus* and *P*. *marmoratus*. Moreover, partial sequences within the complete sequences were also identical. Because H3 is highly conserved, we speculated that the *E*. *sinensis* H3 amino acid sequence might be the same as *H*. *sapiens* and *M*. *musculus*. Based on the codon preference of *E*. *sinensis*, a pair of primers was designed. H3F1, 5′-ATGGCCCGCACCAAACA-3′; and H3R1, 5′-GGCGCGCTCGCCGCG-3′. The primers were synthesized by Beijing Sunbiotech Co., Ltd (Beijing, P. R. China). Touch-down PCR was performed in an XP Cycler (Bioer, Hangzhou, P. R. China). The program included an initial denaturation at 94°C for 5 min, then 30 cycles (0.5°C lower in each cycle) of denaturation at 94°C for 30 s, annealing at 67°C for 30 s, extension at 72°C for 1.5 min, and then 15 cycles of denaturation at 94°C for 30 s, annealing at 52°C for 30 s, and extension at 72°C for 1.5 min, followed by 10 min at 72°C as a final extension. The products were expected to be 408 bp.

### PCR amplification, cloning and sequencing of the H4 coding sequence in *E*. *sinensis*

A pair of degenerate primers (H4F1, 5′-ATGACCGGCCGCGGNAAG-3′, H4R1, 5′-GCCACCGAAGCCRTANARNGT-3′) was designed according to the highly conserved sequences of the full-length histone H4 of *Litopenaeus vannamei* (P83865.2)—also in the Decapoda—and several model organisms such as *H*. *sapiens* (NP_001029249.1), *M*. *musculus* (NP_783583.1), *Drosophila sechellia* (EDW56468.1). The PCR program ran as follows: 94°C for 5 min, 30 cycles of touchdown program (94°C for 30 s, 65°C for 30 s, 72°C for 1.5 min, followed by 0.5°C decrease of the annealing temperature per cycle), followed by 15 cycles at 94°C for 30 s, 50°C for 30 s, 72°C for 1.5 min, and a final extension at 72°C for 10 min. The products were expected to be 309 bp.

The PCR products were separated using 1.0% agarose gels, and visualized under 312 nm ultraviolet (UV) light using a Gel Imaging Analysis System (Beijing Biotechnology Co., Ltd). The corresponding bands were excised and extracted with an Agarose Gel DNA Recovery kit (Beijing Biotechnology Co., Ltd., Beijing, P. R. China), which was then cloned into a pMD-19T cloning vector (Takara). The ligation product was then transferred to *Escherichia coli* DH5α for blue–white screening. The positive recombinant clones were sent to Sangon Biotech, Shanghai, China for sequencing.

### Multiple sequence alignment

Multiple sequence alignment of amino acid sequences was performed using ClustalX software.

### Obtaining free sperm cells

To obtain free spermatozoa from *E*. *sinensis*, we dissected out the seminal vesicles and cut them into pieces manually in Ca^2+^-free artificial seawater (Ca^2+^-FASW; containing 475 mM NaCl, 12 mM KCl, 30 mM MgCl_2_, 20 mM Tris, pH 8.2) [21]. After standing for a few minutes, the supernatants were discarded and the precipitates (containing spermatophores and free sperm cells released from spermatophores) were reserved. Then the accessory glands were homogenized in Ca^2+^-FASW and centrifuged at 10000 rpm for 10 min; the supernatants contained accessory gland proteins. These were added to the previous precipitate (containing spermatophores), after standing for 20 min, and the samples were centrifuged at 500 rpm for 10 min with the supernatants containing sperm cell suspensions. The free spermatozoa were collected by centrifugation at 6000 rpm for 10 min. Then, they were resuspended in Ca^2+^-FASW and centrifuged again. These steps were repeated twice to obtain clean spermatozoa. All procedures were performed at 4°C. The spermatozoa were then used for extracting nuclear proteins.

### Extraction of sperm nuclear proteins and western blot analysis

Nuclear proteins from *E*. *sinensis* sperm were extracted using nuclear and cytoplasmic extraction kits (Beijing ComWin Biotech Co., Ltd. Beijing, P. R. China), according to the manufacturer’s protocol. Following 15% sodium dodecyl sulfate–polyacrylamide gel electrophoresis (SDS-PAGE), proteins were transferred onto polyvinylidene difluoride membranes. Nonspecific binding was blocked at 37°C for 2 h in Tris-buffered saline with Tween-20 (TBST), containing 5% skimmed milk. The membranes were then incubated with an anti-histone H3 antibody (1:5000 dilution) or an anti-histone H4 antibody (1:1000 dilution) overnight at 4°C. The membranes were then washed with TBST and incubated with an HRP conjugated anti-rabbit antibody (1:400 dilution) or anti-mouse antibody (1:400 dilution) at 37°C for 1 h. The membranes were stained using enhanced chemiluminescence (ECL) reagents (Beijing Biotopped Science & Technology Co. Ltd., Beijing, P. R. China). Imaging was carried out using the Chemi Doc XRS+ system with Image Lab Software (Bio-Rad, Hercules, CA, USA).

### Immunofluorescence

Tissue samples from male *E*. *sinensis* were fixed in 4% paraformaldehyde in Tris-buffered saline (TBS, 140 mM NaCl, 20 mM Tris-HCl, pH 7.6) for 48 h. They were then dehydrated in 30% sucrose and embedded in Optimal Cutting Temperature formulation OCT compound (Sakura Finetek Japan Co., Ltd., Tokyo, Japan). Frozen sections were cut at 5 μm thickness and placed on lysine-coated glass slides. The sections were washed in TBS, treated with Quick Antigen Retrieval Solution (Beyotime, Jiangsu, P. R. China) for 5 min, and permeabilized with 0.5% Triton X-100 in TBS, for 20 min at room temperature. Nonspecific antibody binding was blocked by incubation in 5% normal goat serum in tris-buffered saline with Triton X-100 (TBST) for 2 h at 37°C. The sections were then incubated overnight at 4°C in anti-histone H3 antibody (1:1000 dilution) or anti-histone H4 antibody (1:200 dilution). They were then treated with goat polyclonal secondary antibody to rabbit IgG-Fc (conjugated with DyLight 488; 1:300 dilution) or goat anti-mouse IgG H&L (conjugated with Cy3; 1:200 dilution) (Abcam) as a secondary antibody, for 1 h at 37°C, and then washed in TBS before incubation with 4′,6-diamidino-2-phenylindole dihydrochloride (DAPI). Samples were mounted in Antifade Mounting Medium (Beyotime) and observed using an Olympus FV1000-IX81 laser scanning confocal microscope (Olympus, Tokyo, Japan) and images were acquired with FV10-ASW software (Olympus). Negative control sections were processed similarly but were incubated with TBS in place of the primary antibody.

### Colloidal gold labeling for transmission electron microscopy

Tissue fragments were fixed in 4% paraformaldehyde and 1% glutaraldehyde in 0.01 M phosphate-buffered saline (PBS), pH 7.4, at 4°C for 4 h, then washed with 0.01 M PBS to remove the fixative. Samples were then dehydrated in a graded ethanol series and finally embedded in LR White resin (London Resin Co. Ltd., UK) overnight, then changed to fresh LR White, and transferred to gelatin capsules and polymerized at 60°C for 48 h. Ultrathin sections (70 nm), collected on gold grids, were blocked in 0.01 M PBS containing 0.05 M glycine with 1% BSA for 2 h and then incubated overnight with anti-histone H3 antibody (1:80) or anti-histone H4 antibody (1:40) (diluted in 0.01 M PBS, pH 7.4, 0.1% BSA) at 4°C. Following washes, the grids were then incubated with goat anti-rabbit IgG H&L (conjugated with 10 nm gold particles) or Goat Anti-Mouse IgG H&L (conjugated with 20 nm gold particles; diluted 1:50 in 0.01 M PBS, pH 7.4, with 1% polyethylene glycol and 0.02 M NaN_3,_ Abcam) at room temperature. The grids were then washed three times with 0.01 M PBS, followed by three washes in distilled water, and then stained with uranyl acetate. They were examined using a JEM-100SX transmission electron microscope at 75 kV (NEC, Tokyo, Japan). The primary antibody was omitted in controls.

### Quantitative evaluation of gold particle distribution

For each of the two antibodies, three typical cell types (spermatocytes, spermatozoa and testicular somatic cells) were chosen to record the concentrations of gold labeling. The areas of nuclei were measured using Image-Pro Plus 5.1 software (Media Cybernetics, Silver Spring, MD, USA), and the results were expressed as the number of gold particles per μm^2^. Statistical significance of differences in labeling was assessed using a one-way analysis of variance (ANOVA).

### Quantitative analysis of mRNAs for histones H3 and H4 at different stages

The mRNA expression patterns of histones H3 and H4 at different stages were determined by quantitative real-time PCR. Total RNA was isolated from testes of *E*. *sinensis* at different development stages. Genomic DNA elimination, cDNA synthesis and SYBR Green RT-PCR assays were performed using PrimeScript RT reagent kits with a gDNA eraser (Takara) according to the manufacturer’s protocol. A pair of H3 primers: H3RTF, 5′-CAGGATTTCAAGACCGATCTC-3′ and H3RTR, 5′-GATGTCCTTTGGCATGATGG-3′, was used to amplify a product of 146 bp. A second pair of H4 primers: H4RTF, 5′-AAGGTTCTTCGGGACAACAT-3′ and H4RTR, 5′-GGTCTCTTCGTAGATGAGCC-3′ was used to amplify a product of 104 bp. A third pair of 18S rRNA primers: 18S rRNAF, 5′-TTAAAGGAATTGACGGAAGGG-3′, and 18S rRNAR, 5′-GAATTAACCAGACAAATCGCTC-3′, was used to amplify a 181 bp fragment as the internal control. Each 10 μL reaction contained 5 μL of 2 × SYBR Premix Ex Taq II, 0.5 μL forward primer, 0.5 μL reverse primer, 2 μL of water, and 2 μL of template cDNA. All real-time reactions were run in the CFX connect system (Bio-Rad). The PCR program was 95 °C for 1 min; 40 cycles of 95 °C for 10 s, 58 °C for 30 s, and 72 °C for 10 s; and 65 °C for 5 s.

The relative expression levels of histones H3 and H4 were calculated using the comparative Ct method. 18s rRNA was used as an internal control, and the mean of the mature crab controls was used as the calibrator. Briefly, the Ct value of the internal control was subtracted from the Ct value of each sample (ΔCt). Then the value of the calibrator was subtracted (ΔΔCt). Finally, the arithmetic calibrator (2^−ΔΔCt^) was used to calculate the relative expression levels of histones H3 and H4. All data are shown as the mean ± standard deviation (SD) of relative mRNA expression. The mean results were compared using Student’s *t* tests, and p<0.05 was assumed significant.

## Results

### Cloning and sequencing of *E*. *sinensis* histone H3 and protein sequence alignment

**Fig 1:**
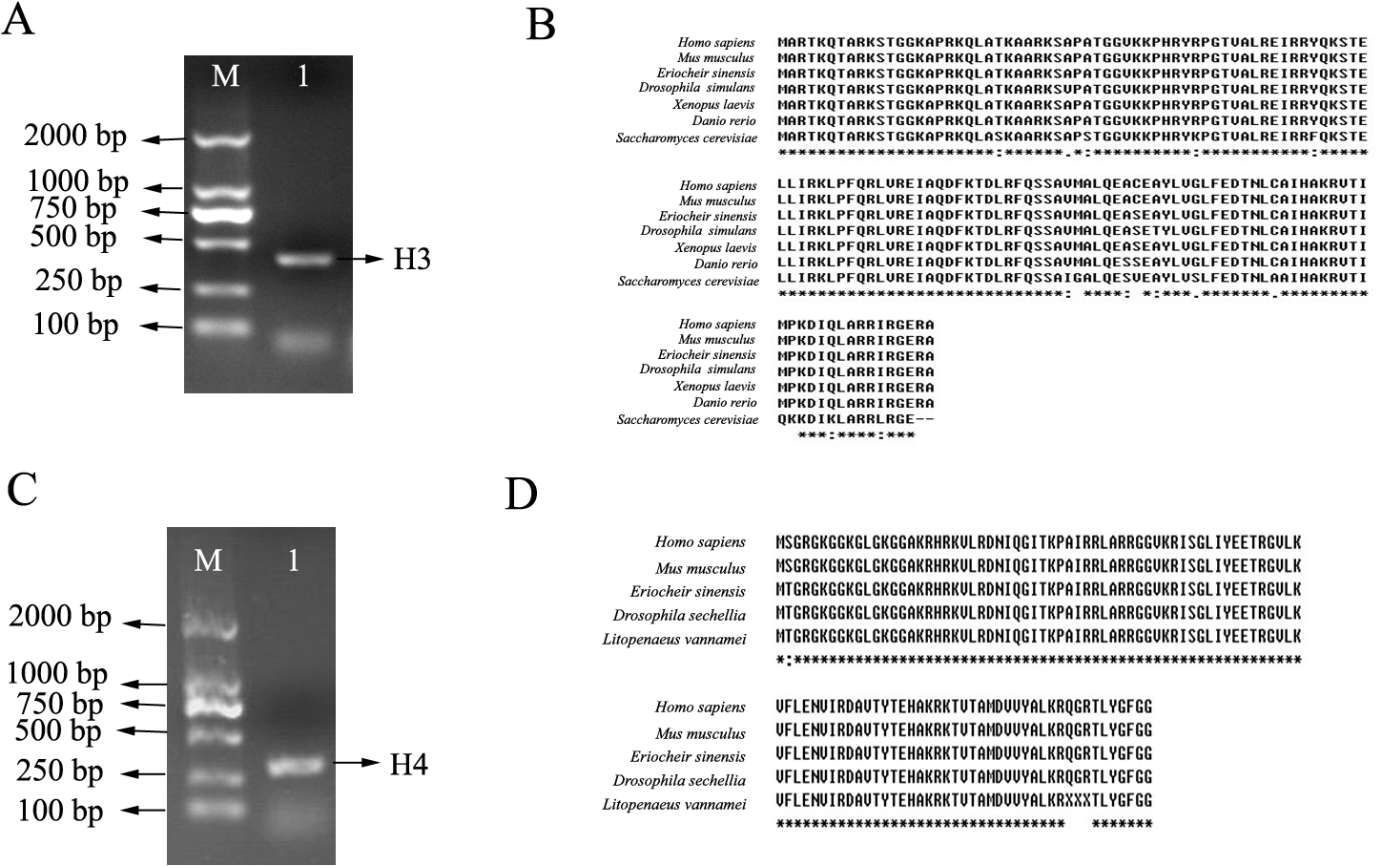
Electrophoretic result of PCR products and multiple alignment of amino acid sequences. (A) The PCR product for H3. M, DM2000 DNA marker; 1, PCR product of *E*. *sinensis* H3. (B) Multiple alignment of deduced H3 amino acid sequence in *E*. *sinensis* with those of other species: *Homo sapiens*, *Mus musculus*, *Drosophila simulans*, *Danio rerio*, *Xenopus laevis*, *Saccharomyces cerevisiae*. (C) The PCR product for H4. M, DM2000 DNA marker; 1, PCR product of *E*. *sinensis* H4. (D) Comparison of deduced amino acid sequence of *E*. *sinensis* histone H4 with other peptides. Symbol key: *the same amino acid; “:” amino acid sequence with strong similar property; “.” amino acid sequence with weak similar property.

A band of about 408 bp was obtained from *E*. *sinensis* testes DNA by PCR with primers (H3F1 and H3R1) that were designed based on the high conservation of histone H3 sequence and codon preference of *E*. *sinensis* through 1% agarose gel electrophoresis (Fig 1A). The band was gel-purified and cloned into a pMD-19T cloning vector. The ligation product was then transferred to *E*. *coli* DH5α for blue–white screening. The positive recombinant clones were sent to Sangon Biotech Co., Ltd, Shanghai, China for sequencing.

The nucleotide sequence and the deduced amino acid sequence are shown in S1 Fig. Multiple sequence alignment of the amino acid sequences of the putative H3 peptides of *E*. *sinensis* with other species was performed using ClustalX software, including *H*. *sapiens*, *M*. *musculus*, *Drosophila simulans* (BAA20144.1), *Danio rerio* (NP-001104686.1), *Xenopus laevis* (AAA49765.1) *Saccharomyces cerevisiae* (AAS64349.1). There was no difference in the amino acid sequence of histone H3 between *E*. *sinensis* and *X*. *laevis*, or only a single amino acid difference when *E*. *sinensis* was compared with *H*. *sapiens*, *M*. *musculus*, and *D*. *rerio* (Fig 1B). Taken together, these findings confirm that the cloned gene encoded *E*. *sinensis* histone H3.

### Cloning and sequencing of *E*. *sinensis* histone H4 and protein sequence alignment

An amplicon of 309 bp was obtained from *E*. *sinensis* testes DNA by PCR with degenerate primers that were designed based on comparing the structures of histone H4 of *L*. *vannamei* and *H*. *sapiens*, *M*. *musculus*, *D*. *sechellia* (Fig 1C). The band was gel-purified, cloned into a pMD-19T cloning vector and sequenced.

The nucleotide sequence and deduced amino acid sequence of histone H4 of *E*. *sinensis* are shown in S2 Fig. The multiple sequence alignment of the amino acid sequences of the putative H4 peptides of *E*. *sinensi*s with that of *L*. *vannamei* (P83865.2), *H*. *sapiens* (NP_001029249.1), *M*. *musculus* (NP_783583.1) and *D*. *sechellia* (EDW56468.1) confirmed that the cloned gene encoded *E*. *sinensis* histone H4, as there is only one amino acid difference between *E*. *sinensis* and *H*. *sapiens* and *M*. *musculus*. Moreover, *E*. *sinensis* shared the same sequence with *D*. *sechellia* (Fig 1D).

### Validity of antibodies for *E*. *sinensis*

Anti-histone H3 and anti-histone H4 antibodies were raised against residue 100 to the C-terminus of human histone H3 and residue 50 to the C-terminus of human histone H4, respectively. We compared the H3 and H4 amino sequences with human immunogen sequences, which showed similarities of 99% and 100% respectively (Fig 1B and 1D). Thus, it was valid to use these commercial antibodies.

### Identification of histones H3 and H4 in sperm nuclei of *E*. *sinensis*

**Fig 2:**
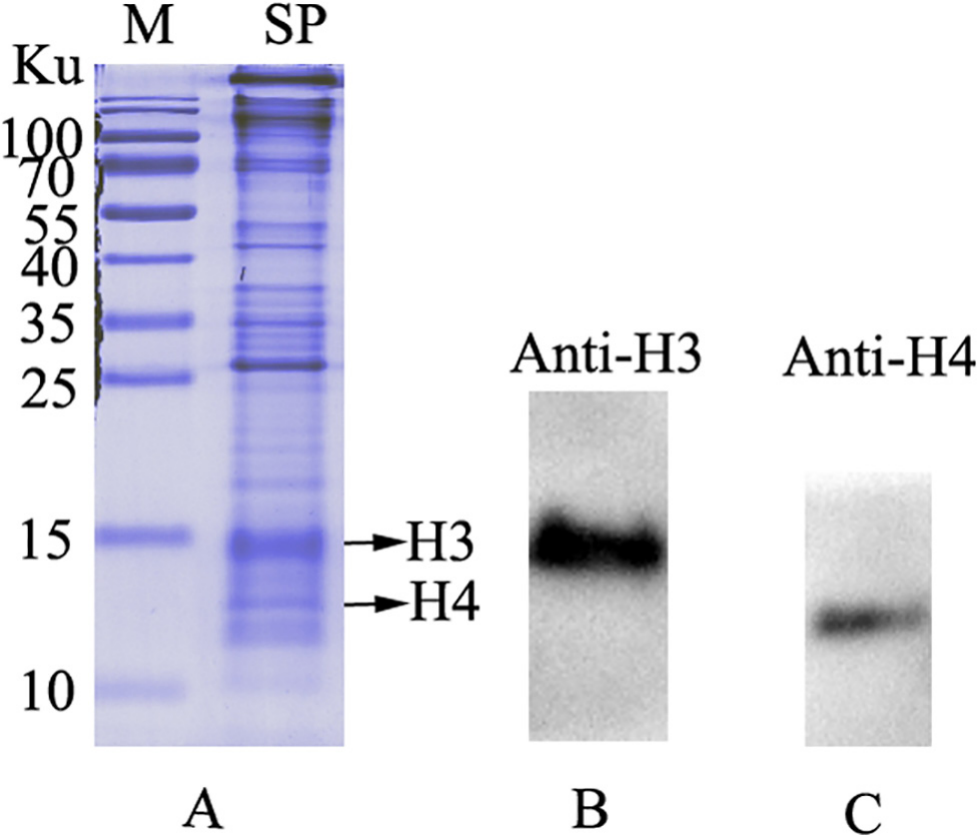
Identification of histones H3 and H4 in sperm nuclei of *E*. *sinensis*. (A) Sperm nuclear proteins separated by 15% SDS-PAGE. Individual arrows show histones H3 and H4 respectively. (B) Western blot of sperm nuclear proteins labeled with an anti-histone H3 antibody. (C) Western blot of sperm nuclear proteins labeled with an anti-histone H4 antibody. Key: M, marker protein; SP, sperm nuclear proteins.

Western blotting was performed to determine whether histones H3 and H4 were expressed in the sperm nuclei. Following SDS-PAGE of nuclear proteins extracted from *E*. *sinensis* spermatozoa, the anti-histone H3 antibody recognized a band of about 15 Ku, and the anti-histone H4 antibody recognized a band of about 11 Ku (Fig 2). Thus, western blot analysis confirmed the presence of histones H3 and H4 in the sperm nuclei of *E*. *sinensis*.

### Temporal and spatial expression profiles of histones H3 and H4 during *E*. *sinensis* spermatogenesis

#### Spermatogonia

**Fig 3:**
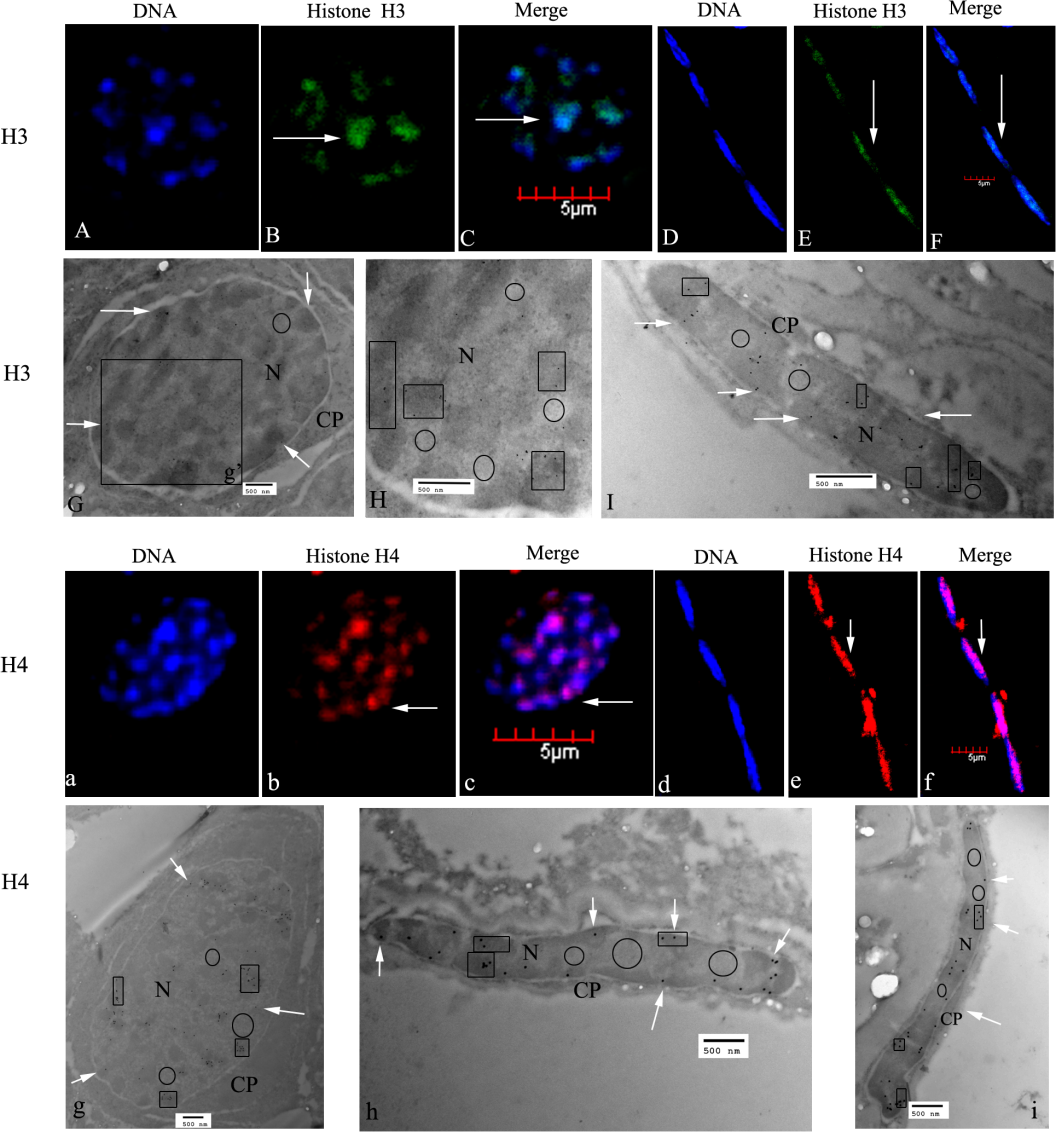
Localization of histones H3 and H4 in spermatogonia of *E*. *sinensis*. (A–C) Immunofluorescent localization of histone H3 in spermatogonia. DNA was stained with DAPI (blue), and histone H3 was visualized with a goat polyclonal secondary antibody to rabbit IgG-Fc conjugated to DyLight 488 (green). (D–F) Immunofluorescent localization of histone H3 in testicular somatic cells. The nuclei show histone H3 staining (see arrows in E, F). (G) Ultrastructural localization of histone H3. Histone H3 was mainly localized in the nuclei of spermatogonia (see arrows in G). (H) A magnified part of G (g’); H3 was mainly localized in heterochromatin (see panes). (I) Ultrastructural localization of histone H3 in testicular somatic cells. H3 was mainly found in nuclear heterochromatin (see arrows and panes). (a–c) Immunofluorescent localization of histone H4 in spermatogonia. H4 was mainly localized in the nucleus (see arrows in b and c). (d–f) Immunofluorescent localization of histone H4 in testicular somatic cells. H4 was mainly found in nucleus (see arrows in e and f). (g) Ultrastructural localization of histone H4 by electron microscopy (EM). H4 was mainly found in the nuclear heterochromatin (see arrows and panes). (h and i) Ultrastructural localization of histone H4 in testicular somatic cells. H4 was mainly distributed in nuclear heterochromatin (see arrows and panes). Key: N, nucleus; CP, cytoplasm. The regions in panes represent heterochromatin, which stains intensely, the circled areas represent euchromatin, which is less intensely stained. Scale bars in immunofluorescent images = 5 μm; those within EM images = 500 nm.

Spermatogonia in *E*. *sinensis* are spherical or elliptical, and are the largest among all cell stages. The nucleus is spherical or oval. Most of the chromatin was scattered, while some was assembled into small patches, either onto the inner surface of the nuclear membrane or randomly distributed (Fig 3A and 3A). Immunofluorescent results showed that histones H3 and H4 were distributed in the nuclei of spermatogonia (Fig 3A–3C and 3A–3C). The nuclei of testicular somatic cells also showed staining for histones H3 and H4 (Fig 3D–3F and 3D–3F). Immunogold labeling demonstrated that histones H3 and H4 were mainly localized in nuclear heterochromatin, which stained intensely (Fig 3G–3H and 3G). The nuclei of testicular somatic cells were also labeled (Fig 3I, 3H and 3I). The cytoplasm of spermatogonia and testicular somatic cells were all unlabeled.

#### Spermatocytes

**Fig 4:**
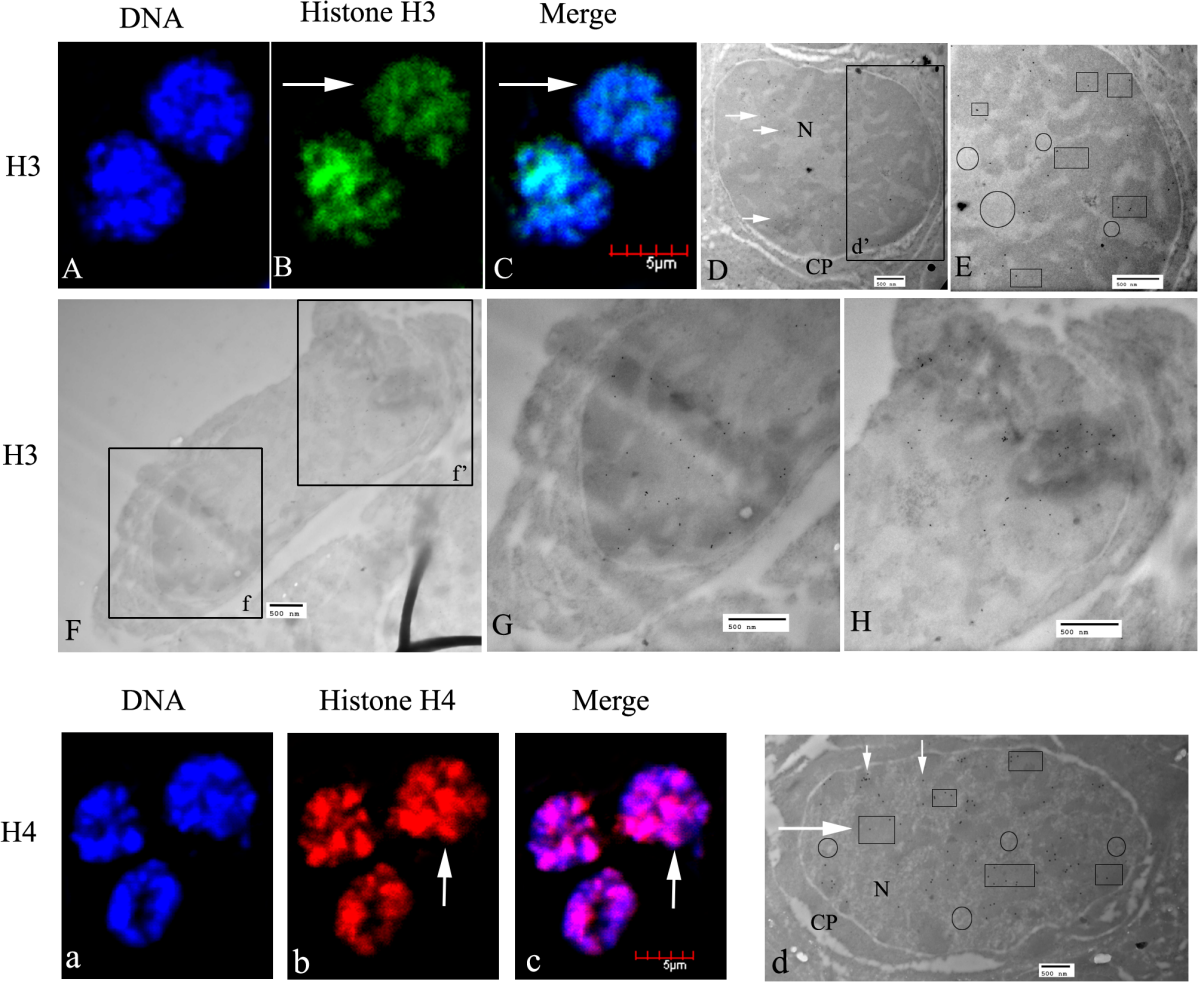
Localization of histones H3 and H4 in spermatocytes of *E*. *sinensis*. (A–C) The nuclei show histone H3 staining (see arrows in B, C), and the chromatin is assembled into patches (A). (D) Ultrastructural localization of histone H3. H3 was mainly distributed in nuclei (arrowheads). (E) A magnified part of D (d′). H3 was mainly distributed in heterochromatin (see panes). (F) A spermatocyte was undergoing meiosis. (G and H) Magnified views of F (f) and F (f′), showing that histone H3 was pulled toward the spindle poles with chromosome separation. (a–c) The nuclei of spermatocytes show histone H4 staining (see arrows in b and c), with the chromatin assembled into patches in (a). (d) Ultrastructural localization of histone H4. In spermatocytes, histone H4 was mainly localized in the nuclear heterochromatin (see arrows and panes in D). Key: N, nucleus; CP, cytoplasm. The regions in panes show heterochromatin, which stains intensely; the circled regions show euchromatin, which is less intensely stained. Scale bars in immunofluorescent images = 5 μm; those in EM images = 500 nm.

Spermatocytes in *E*. *sinensis* are polygonal, and a little smaller than spermatogonia. The nuclei appear spherical or oval, and the chromatin is assembled into patches positioned either around the nuclei or on one side (Fig 4A and 4A). In the spermatocytes, the signals for H3 and H4 were also localized in the nuclei and no labeling was observed in the cytoplasm (Fig 4A–4E and 4A–4D). Fig 4F shows a spermatocyte undergoing meiosis; histone H3 was linked with the chromosomes and was pulled toward the poles.

#### Spermatids and mature spermatozoa

**Fig 5:**
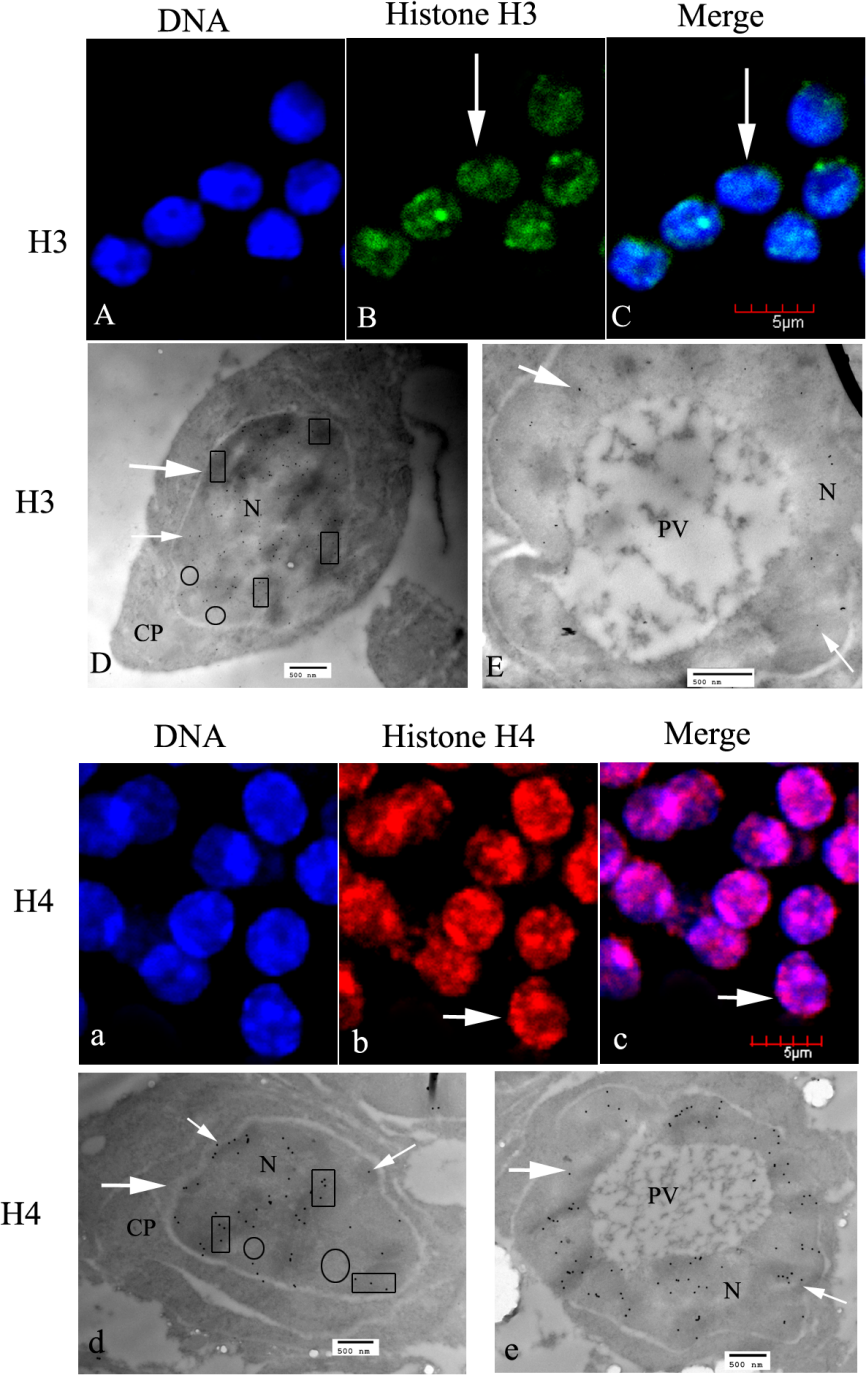
Location of histones H3 and H4 in spermatids of *E*. *sinensis*. (A–C) Immunofluorescent localization of histone H3 in early spermatids. (D) Ultrastructural localization of histone H3 in early spermatids. (E) Ultrastructural localization of histone H3 in intermediate spermatids. (a–c) Immunofluorescent localization of histone H4 in early spermatids. (d) Ultrastructural localization of histone H4 in early spermatids. (e) Ultrastructural localization of histone H3 in intermediate spermatids. Histones H3 and H4 were present in the nuclei throughout these stages (see arrows and panes). Key: N, nucleus; PV, proacrosomal vesicle. The regions in panes show heterochromatin, which stains intensely; the circled regions show euchromatin, which stains less intensely. Scale bars in immunofluorescent images = 5 μm; those in EM images = 500 nm.

**Fig 6:**
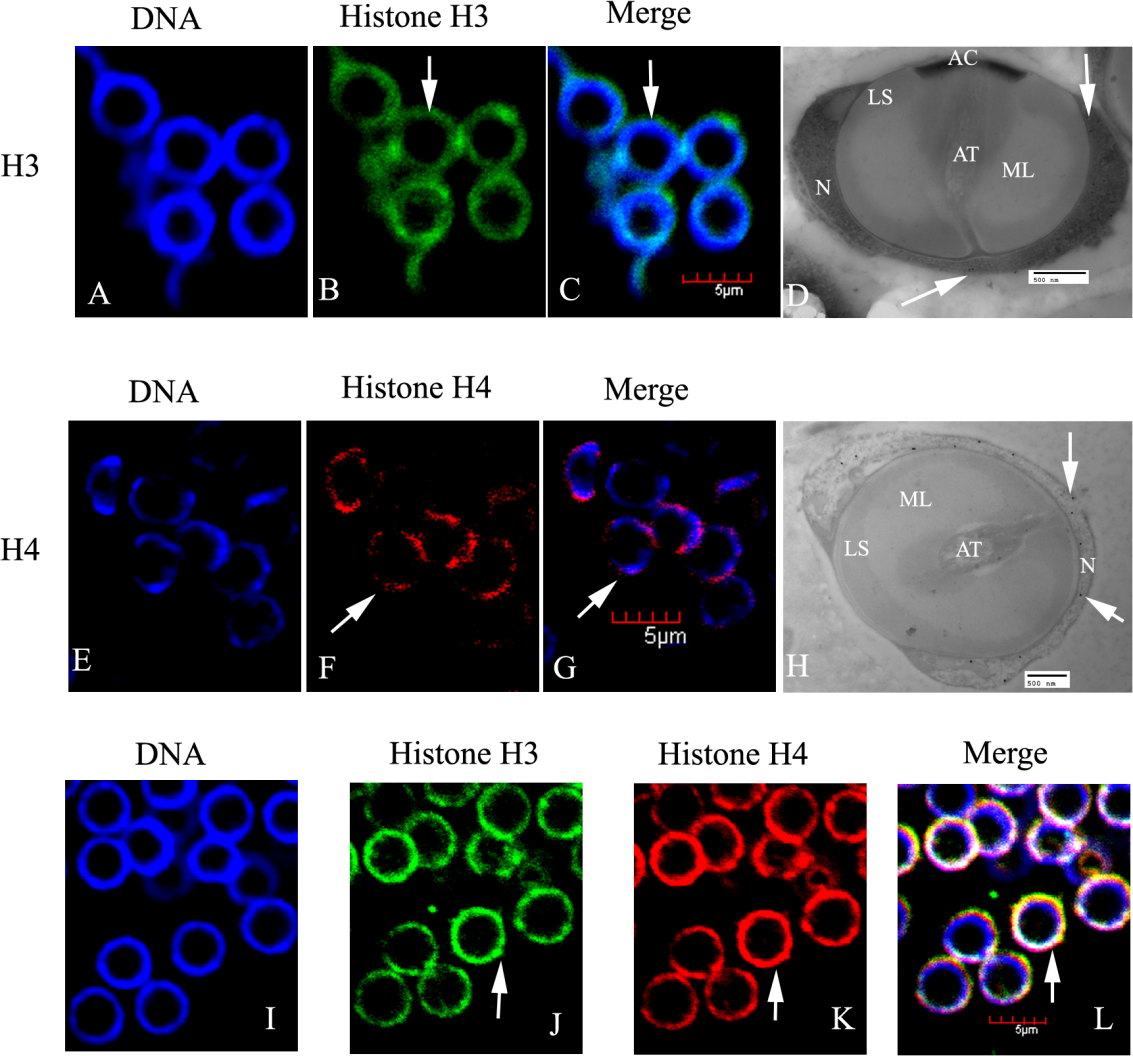
Localization of histones H3 and H4 in spermatozoa of *E*. *sinensis*. (A–C) Immunofluorescent localization of histone H3 in spermatozoa. (D) Ultrastructural localization of histone H3 in spermatozoa. (E–G) Immunofluorescent localization of histone H4 in spermatozoa. (H) Ultrastructural localization of histone H4 in spermatozoa. (I–L) Dual H3 and H4 immunofluorescent staining. Histones H3 and H4 were present in the nuclei of mature spermatozoa (arrows). Key: N, nucleus; AC, apical cap; AT, acrosomal tubule; ML, middle layer; LS, lamellar structure. Scale bars in immunofluorescent images = 5 μm; those in EM images = 500 nm.

The spermatids are spherical or oblong. Chromatin of the early spermatid was filamentous and well diffused. Histones H3 and H4 were mainly distributed in the nuclei at this stage (Fig 5A–5D and 5A–5D). At the mid spermatid stage, a large proacrosomal vacuole (PV) closed to the nucleus was formed; histones H3 and H4 immunoreactive sites could be seen in the nucleus. No labeling was seen in the PV (Fig 5E and 5E). At the late spermatid stage, spermatozoa of *E*. *sinensis* became mature, with the formation of a cup-shaped nucleus and a well-developed acrosome. At this time, histones H3 and H4 were still concentrated in the nucleus (Fig 6A–6C and 6E–6G), but the apical cap, acrosomal tubule, middle layer, and lamellar structure were all unlabeled (Fig 6D and 6H). Dual H3 and H4 immunofluorescent were shown in Fig 6I–6L, which demonstrated that histone H3 were basically colocalized with H4.

### Quantitative changes of histones H3 and H4 in nuclear distribution

**Fig 7:**
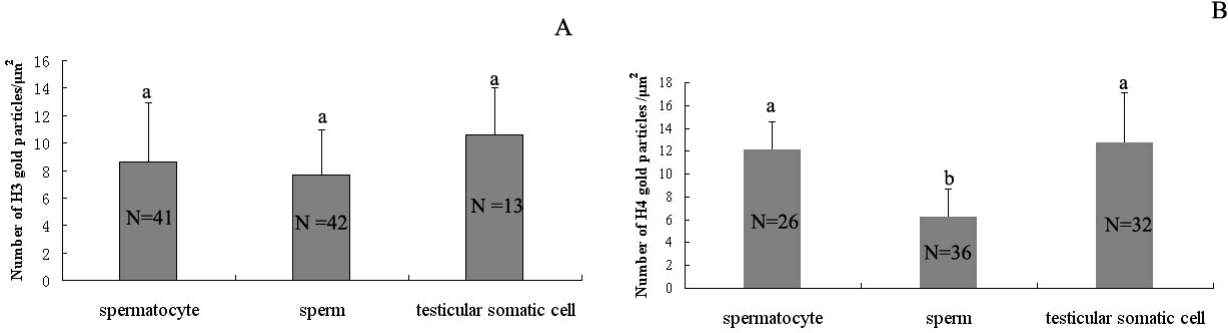
Patterns of nuclear labeling for histones H3 and H4 at different stages. Vertical bars represented the mean ± SD of the numbers of gold particles. (A) The nuclear labeling density of histone H3 did not show significant differences among spermatocytes, spermatozoa or testicular somatic cells. (B) Histone H4 labeling in spermatozoa decreased significantly compared with spermatocytes and somatic cells. Different letters above the error bars indicate significant differences (p<0.05).

The patterns of nuclear labeling for histones H3 and H4 at different periods are shown in Fig 7. The nuclear labeling density of histone H3 did not change significantly among spermatocytes, spermatozoa and testicular somatic cells. The nuclear labeling density of histone H4 in spermatozoa was significantly lower than in spermatocytes or testicular somatic cells (p<0.05), and H4 labeling was not significantly different between spermatocytes and testicular somatic cells.

### Quantitative analysis of mRNA expression levels of *E*. *sinensis* histones H3 and H4 at different stages

**Fig 8:**
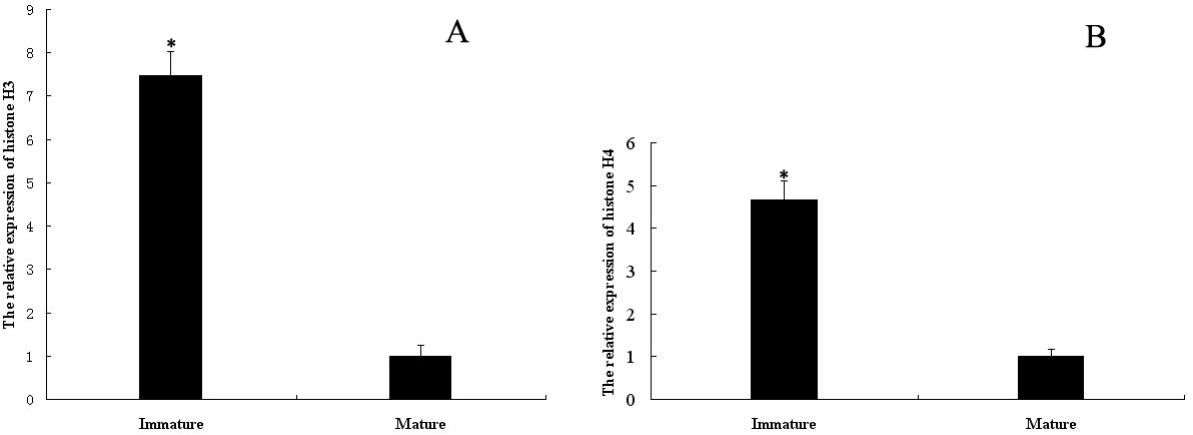
*E*. *sinensis* mRNA expression levels of histones H3 and H4 during spermatogenesis. Most germ cells in the testes of immature crabs were undergoing mitosis and meiosis, while those of mature crabs were undergoing spermiogenesis. Data are shown as the mean ± SD (n = 4). One star indicates significant difference compared with control (p<0.05). (A) The relative expression level of histone H3 mRNA; (B) The relative expression of histone H4 mRNA.

The relative mRNA expression levels of histones H3 and H4 at different stages were analyzed by quantitative real-time PCR, using 18S rRNA as the internal control. The major germ cells in testes of immature crabs were spermatogonia and spermatocytes, while in the mature crabs they were mainly spermatids and spermatozoa. The variations in mRNA levels of histones H3 and H4 were the same among the various stages within these groups. However, the mRNA expression levels of histones H3 and H4 in the testes of immature crabs were significantly higher than in the mature crabs (p<0.05; Fig 8).

## Discussion

Histones are the basic structural proteins that make up eukaryotic chromosomes, and play important roles in maintaining and controlling chromosomal structure [22].

The encoding genes of histones have no introns, so we extracted genomic DNA to amplify H3 and H4 cDNA encoding sequences. Considering the high conservation of histones and codon preference of *E*. *sinensis*, we designed two pair of primers and obtained PCR products with expected sizes of 408 and 309 bp. The result of multiple alignments was consistent with the fact that H3 and H4 proteins are highly evolutionarily conserved [23, 24].

A number of studies have been carried out to understand the characteristics of histones in the sperm nuclei of decapod crustaceans. However, there have been some conflicting results from the studies regarding the presence of SNBPs in crustaceans. With specific histochemical staining methods, early researchers thought that the sperm nuclei chromatin of crabs was uncondensed because they contained no histones or basic proteins. For example, a large quantity of nucleoproteins in spermatids of *E*. *analogy* was gradually reduced during spermatogenesis and there were considered to be nearly no histones or protamines in the late stage in sperm nuclei [15]. Similar results were recorded in studies on *Metapenaeus ensis* and *Scylla serrata* [25, 26]. In addition, Kleve [16] found that no basic protein was present in the sperm nuclei of *S*. *ingentis*. Even a graduate student in our laboratory thought that the mature sperm nuclei of *Fenneropenaeus chinensis* did not contain H4 [27]. Later, Chevaillier found that histones migrated from the nucleus to the acrosomal vesicle in *E*. *bernhardus*. Because of a lack of quantitative assessment, weak staining may have led to an oversight regarding the presence of histones in decapod sperm nuclei [28]. Recent studies by Kurtz *et al* [18, 19] have shown that mature spermatozoa of some crab species do have histones.

Because of these conflicting data regarding the presence of SNBPs in the mature spermatozoa of crustaceans, we investigated the two most conserved histones: H3 and H4. Our western blot analyses of sperm nuclear proteins using antibodies specific for H3 and H4 proteins showed positive immunoreactivity. This indicated that mature sperm nuclei of *E*. *sinensis* contain histones H3 and H4. In addition, our immunofluorescent staining and immunoelectron microscopy demonstrated that H3 and H4 were present throughout the whole of spermatogenesis, and in testicular somatic cells. These data contradict previous studies claiming that nuclei lose all histones in the most advanced stages of spermiogenesis, and that SNBPs are absent in decapods [29]. Sellos and Legal [30] reported that the mature sperm nuclei of *Palaemon serrifer* also have some sperm-specific basic proteins. Nevertheless, our present results are in accordance with recent reports in *P*. *pelagicus*, *M*. *brachydactyla* and *Cancer* crabs [18–20], that *E*. *sinensis* sperm nuclei retain some SNBPs. These data may provide some explanation of why the sperm chromatin of *E*. *sinensis* is highly decondensed. While histones H4 and H3 are conserved in other species, such as the rabbit, trout, rooster, and mouse, histones are completely replaced by transition proteins and protamines leading to nuclear chromatin condensation [31–35]. However, this replacement happens less often or not at all in *E*. *sinensis*. In addition, the nuclear labeling density of histone H3 did not differ significantly between different stages of spermatogenesis. Although the presence of histone H3 variants in spermatozoa of *E*. *sinensis* has not been reported, this possibility could not be excluded. The polyclonal anti-H3 antibody was raised against residues 100 to the C-terminus of human histone H3, which are highly conserved between H3 variants [36]. Therefore, the availability of the antibody shouldn’t be markedly changed by the minor alteration of the histone H3 molecule [34]. The patterns of labeling for histone H3 seems to be consistent with such a case, as which did not have significant change in our present study. However, histone H4 in mature spermatozoa displayed a decreasing tendency compared with spermatocytes and somatic cells. This might be related to post-translational modifications such as acetylation, since in *Cancer* the histone H4 is highly acetylated. These modifications might influence the interactions of histones either with anti-histone antibodies or with DNA, And also, it leads to the unfolding of nucleosome arrays [37]. Analysis of the amounts of histones H3 and H4 might suggest that the structural units formed by DNA–histone complexes are not the same as nucleosomes, because the amounts of core histones in nucleosomes are equal [38]. Despite the disproportionate quantity of histones H3 and H4 in spermatozoa of *E*. *sinensis*, this phenomenon is not exclusive to this species. Studies on the amphibian *X*. *laevis* showed that the content of histones H3 and H4 in sperm nuclei are identical to those of somatic cells, but that they have lower levels of histones H2A and H2B, and are completely lacking H1 [39–41]. In this case, the sperm nucleus appears condensed because the H2A–H2B dimers are substituted by other basic proteins (Sp1-Sp6) [42, 43]. Studies on *Homarus vulgaris* [44], *Homarus americanus* and *Nephrops norvegicus* [45] showed that sperm have fine granular nuclear material, which is related to the maintenance of basic histones. Based on these analyses, we speculate that the conservation of histones might have some relationship to the lack of condensation of sperm chromatin of *E*. *sinensis*. It has been proposed that because spermatozoa of decapod crustaceans do not need to travel far to fertilize, there is no need to pack the chromatin tightly to protect the DNA from environmental damage [46], so the chromatin remains arranged loosely.

The mRNA expression levels of histones H3 and H4 in immature crabs in the rapid development stage were higher than in mature crabs. The testes of immature crabs mainly include spermatogonia and spermatocytes undergoing mitosis and meiosis. Therefore, histones H3 and H4 might be synthesized in high amounts during this period.

In summary, we have cloned the genes encoding histones H3 and H4 of *E*. *sinensis*, and confirmed that *E*. *sinensis* sperm nuclei contain histones H3 and H4, albeit with unusually uncondensed chromatin. Moreover, we have discussed the potential relationship between the conservation of histones and uncondensed sperm chromatin. This is the first report that the mature sperm nucleus of *E*. *sinensis* contains histones H3 and H4. The results broaden our understanding of the uncondensed sperm chromatin of *E*. *sinensis*.

## Supporting Information

S1 Fig The nucleotide sequence and decuced amino acid sequence of histone H3 in *E*. *sinensis*. (TIF)

S2 Fig The nucleotide sequence and decuced amino acid sequence of histone H4 in *E*. *sinensis*. (TIF)
