# Supplementary figures and images for: Cloning and Functional Analysis of Histones H3 and H4 in Nuclear Shaping during Spermatogenesis of the Chinese Mitten Crab, Eriocheir sinensis
Source: PLoS One. 2015 May 19;10(5):e0126623. doi: 10.1371/journal.pone.0126623 (PMC4438001; doi:10.1371/journal.pone.0126623)

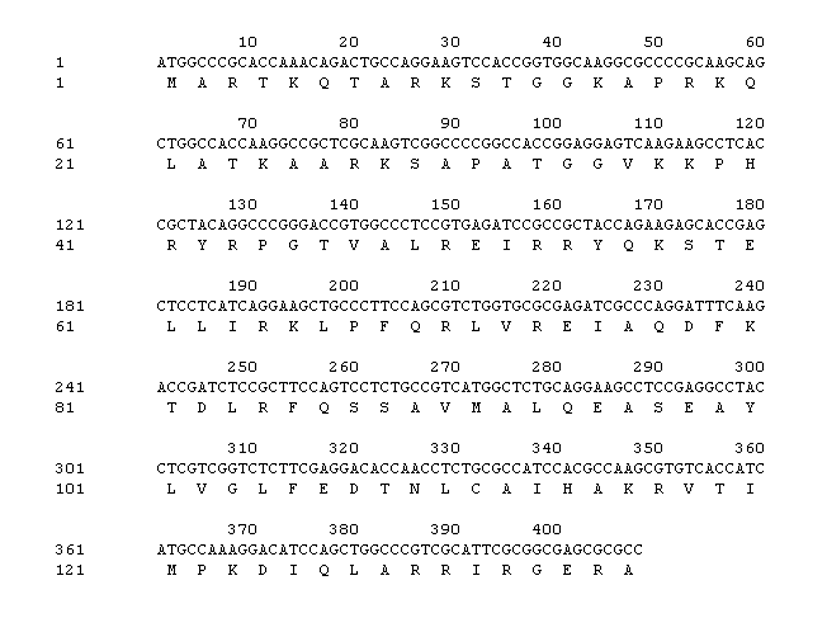

Supplement: S1 Fig — (TIF) [file pone.0126623.s001.tif]

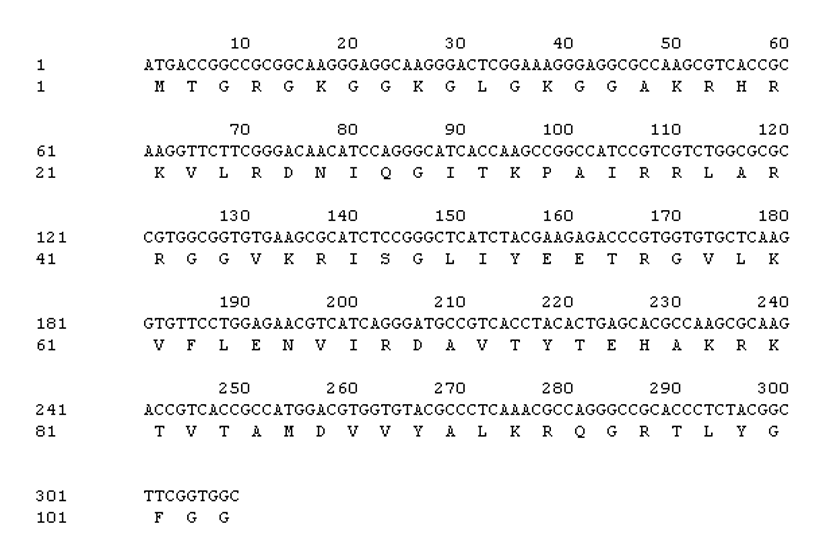

Supplement: S2 Fig — (TIF) [file pone.0126623.s002.tif]
